# An in depth review of body shaming phenomenon among adolescent: Trigger factors, psychological impact and prevention efforts

**DOI:** 10.4102/sajpsychiatry.v30i0.2341

**Published:** 2024-11-14

**Authors:** Fitrio Deviantony, Yeni Fitria, Rondhianto Rondhianto, Ni Komang T. Pramesuari

**Affiliations:** 1 Department of Mental Health, Faculty of Nursing, Jember University, Jember, Indonesia; 2 Department of Medical Surgical, Faculty of Nursing, Jember University, Jember, Indonesia

**Keywords:** body shaming, adolescence, nurse, students, education

## Abstract

**Background:**

Body shaming, a pervasive issue, has severe psychological and societal repercussions, particularly for early adolescents. This study addresses the gap in understanding body shaming in smaller urban settings such as Jember City, often overlooked in favour of larger metropolitan areas.

**Aim:**

The study aimed to investigate the psychological effects, trigger factors and potential preventive measures of body shaming among junior high school students in Jember City.

**Setting:**

The study was conducted in junior high schools in Jember City, East Java, Indonesia.

**Methods:**

An observational, cross-sectional design with both quantitative and qualitative approaches was used. Data were collected through self-administered questionnaires and in-depth interviews. The quantitative analysis was performed using the Spearman’s rank test.

**Results:**

The study included 320 adolescents aged between 12 and 15 years. Of these, 6.3% were 12 years old, 31.2% were 13, 31.6% were 14, and 30.9% were 15. Additionally, 56.2% of the participants were female. The majority (95%) identified as Muslim, and 72.8% had parents with bachelor’s degrees. Parental occupations ranged from private business to civil service. In terms of body mass index, 34.7% were classified as very thin, 18.1% as thin, 40.9% as normal, and 3.1% as fat or obese. Body shaming was widespread, with 73.1% criticized for their clothing, 59.9% for their speech, and 66.7% compared to others. The main sources of body shaming were family, peers, media, and personal insecurities. There was also a significant correlation between body shaming and stress (ρ = 0.404, *p* < 0.01).

**Conclusion:**

Body shaming affects mental health, particularly among adolescents. It stems from societal norms and media perpetuation.

**Contribution:**

This study provides insights into body shaming in smaller urban settings, highlighting the need for targeted prevention efforts to mitigate its effects and promote healthier self-esteem and body image.

## Introduction

### Social value

Adolescence is a critical period of transition between childhood and adulthood, characterised by significant changes in the body, mind and emotions. This stage involves synchronous biochemical, cognitive, psychological and emotional developments that create a pathway from childhood to maturity.^1^ Given these substantial transformations, adolescents are particularly susceptible to social and psychological challenges, such as body shaming. Body shaming, defined as the act of criticising someone’s physical appearance, can have severe psychological repercussions, including stress, anxiety, depression and low self-esteem.^2,3^ Research indicates that body shaming incidents are prevalent among adolescents and can profoundly affect their mental health and social well-being.^4,5^ Understanding the dynamics of body shaming among adolescents is essential for developing effective preventive measures and support systems to enhance their psychological resilience and overall well-being.

### Scientific value

While body shaming has been extensively studied in larger metropolitan areas, there is a notable gap in understanding this phenomenon in smaller urban settings such as Jember City. Existing studies highlight the prevalence of body shaming among adolescents globally, but specific insights into its occurrence and impact in smaller cities are limited.^6^ This study aims to address this knowledge gap by investigating body shaming among junior high school students at Jember City, examining its psychological effects, trigger factors and potential preventive measures. By focussing on a smaller urban context, this research provides a unique contribution to the broader understanding of body shaming, offering insights that can inform targeted interventions and policies tailored to similar settings.

### Conceptual framework

The study is grounded in the theoretical framework that adolescence is a period of heightened vulnerability to social comparison and external judgements because of significant physical and psychological changes.^5,7^ This framework posits that adolescents’ self-esteem and body image are heavily influenced by social interactions and cultural norms around beauty and appearance.^8^ The interplay between family influence, peer pressure, media representations and personal insecurities forms the basis for understanding the trigger factors of body shaming.^9,10^ The study employs a mixed-methods approach to capture both the quantitative prevalence and qualitative experiences of body shaming, providing a comprehensive understanding of the issue.

### Aim and objectives

The aim of this study is to investigate the phenomenon of body shaming among junior high school students at Jember City, focussing on its psychological effects, trigger factors and potential preventive measures. The specific objectives are to determine the prevalence of body shaming experiences among adolescents in Jember City, to identify the primary trigger factors contributing to body shaming, to assess the psychological impact of body shaming on adolescents and to propose preventive measures and interventions to mitigate the effects of body shaming. By addressing these objectives, this study seeks to contribute valuable insights into the prevalence and impact of body shaming in smaller urban settings, highlighting the need for targeted strategies to support adolescents’ mental health and well-being.

## Research methods and design

### Study design

This study employed a mixed methods design, integrating qualitative data to supplement quantitative data. The primary approach was an observational cross-sectional design aimed at examining relationships between variables at a specific point in time.^11^

### Setting

The study was conducted in a junior high school setting at Jember, East Java, Indonesia. The population comprised students from various junior high schools within Jember City, a smaller urban environment with diverse socio-economic backgrounds.

### Study population and sampling strategy

The study population included junior high school students aged 12–15 years. Inclusion criteria were students willing to participate, actively enrolled and within the specified age range. Exclusion criteria included students who refused to participate, withdrew from the study or were not attending school regularly. Using Slovin’s formula for sample size calculation, the required sample size was determined to be 320 respondents. The sampling strategy employed was stratified random sampling, dividing the population into strata based on grade levels and then randomly selecting proportional samples from each stratum to ensure representation across the various schools at Jember.

### Data collection

Data were gathered through both primary and secondary sources. Primary data collection involved distributing questionnaires to students from first to third grade, covering demographic information, body shaming experiences and stress levels. The data collection process included three phases: preparation, screening and implementation. Permissions and ethical clearances were obtained before data collection commenced. The body shaming questionnaire included questions adapted from previous studies, while stress levels were measured using the Depression, Anxiety and Stress Scales 42 (DASS-42). The Depression, Anxiety, and Stress Scales (DASS) is a validated tool used in this study to measure the core symptoms of stress, anxiety and depression among the respondents. The DASS provides a comprehensive assessment of these three psychological states, allowing for the identification of the severity and interplay of stress, anxiety and depression symptoms in the context of body shaming. In this article, the DASS is identified as the primary tool for measuring the mental health effects of body shaming on early adolescents.^12^ Both questionnaires underwent validity testing, yielding a validity coefficient of r = 0.413 and a result range of 0.436–0.705. Additionally, reliability tests using Cronbach’s Alpha showed values exceeding 0.60, with a score of 0.918.

### Data analysis

Data analysis was conducted using SPSS 26.0. Descriptive statistics were used to summarise all parameters. Categorical data were presented as frequencies and percentages, while numerical data were summarised using median and mean. The Kolmogorov–Smirnov test was applied to evaluate the normality of continuous variables. Depending on the normality, Pearson’s test was used for normally distributed data, and the Spearman’s rank test was used for non-normally distributed data. Bivariate analysis was carried out to determine the relationship between body shaming and stress levels using the Spearman’s rank test, with a significance level set at *p* < 0.05, this is crucial to understanding the broader mental health implications. It is important to notice that in this context, ‘stress levels’ encompass not only stress but also the closely related symptoms of anxiety and depression. The statistical strength, direction and significance of the results were interpreted accordingly.

### Ethical considerations

Ethical approval for the study was obtained from the Health Research Ethics Committee at the Faculty of Nursing, University of Jember, with approval number 193/UN25.1.14/KEPK/2022. Informed consent was obtained from all participants prior to data collection, ensuring their voluntary participation and confidentiality of their responses.

## Results

The study involved 320 adolescents with diverse sociodemographic backgrounds. The participants’ ages ranged from 12 to 15 years old, with the majority being 14 years old (31.6%) and 13 years old (31.2.%). The gender distribution indicated a slightly higher number of female participants (56.2%) compared to males (43.8%). In terms of religious affiliation, a significant majority identified as Muslim (95.0%), followed by a small number of Christians (4.1%), Catholics (0.6%) and Hindus (0.3%). When considering the educational grade level, participants were fairly distributed across the first (29.7%), second (36.9%) and third grades (33.4%). The body mass index (BMI) categorisation revealed that 34.7% of the participants were classified as very thin, 18.1% as thin and 40.9% had a normal BMI, while both the fat and obesity categories each comprised 3.1% of the participants (Table 1).

**TABLE 1:** Sociodemographic characteristics of participants.

Characteristic	Frequency (*f* = 320)	%
**Age (years)**		
12	20	6.3
13	100	31.2
14	101	31.6
15	99	30.9
**Gender**		
Male	140	43.8
Female	180	56.2
**Religion**		
Islam	304	95.0
Christian	13	4.1
Catholic	2	0.6
Hindu	1	0.3
**Class or grade**		
First grade	95	29.7
Second grade	118	36.9
Third grade	107	33.4
**BMI**		
Very thin	111	34.7
Thin	58	18.1
Normal	131	40.9
Fat	10	3.1
Obesity	10	3.1

BMI, body mass index.

A total of 320 participants provided information about their parents’ educational background and occupation. The majority of parents held a bachelor’s degree (72.8%), while 21.2% had completed senior high school, 5.6% had finished junior high school and a minimal 0.3% had only completed elementary school (Table 2).

**TABLE 2:** Characteristic of participant’s parent.

Demographic data	Frequency (*f* = 320)	%
**Parents’ last education**		
Elementary school	1	0.3
Junior high school	18	5.6
Senior high school	68	21.2
Bachelor’s degree	233	72.8
**Parents’ job**		
Civil servants	72	24.2
Private business	88	33.3
Entrepreneur	71	23.9
Farmers	4	1.3

Other	51	17.2

Regarding parental occupation, private business was the most common job (33.3%), followed by civil servants (24.2%) and entrepreneurs (23.9%). A smaller proportion of parents were farmers (1.3%) or engaged in other types of employment (17.2%).

The phenomenon of body shaming among adolescents is a multifaceted issue, deeply rooted in societal norms and peer interactions. Analysing data from various indicators provides a comprehensive understanding of this pervasive problem. One of the primary aspects of body shaming is commenting on appearance, where a significant 73.1% of adolescents reported being criticised for their dress style and 59.9% experienced criticism regarding their speaking style. Behaviour-based criticism also stands out, with 76.8% of adolescents facing such negativity. Gossip, often overlooked, affects 53.2% of adolescents, revealing its significant role in perpetuating body shaming. The tendency to compare oneself with others physically is another crucial trigger, with 46.1% of adolescents engaging in self-comparison and 66.7% being compared to others by their peers (Table 3).

**TABLE 3:** Distribution of body shaming experience indicators (*N* = 320).

Aspect	Indicator	Low		Medium		Height		Total	
		*F*	%	*F*	%	*F*	%	*F*	%
**Body shaming experience**									
Commenting on appearance	Criticised for how to dress	67	22.6	217	73.1	13	4.4	297	100
	In criticism of style in speaking	89	30.0	178	59.9	30	10.1	297	100
	Criticised for behaviour	58	19.5	228	76.8	11	3.7	297	100
	Gossip about bad things	131	44.1	158	53.2	8	2.7	297	100
Comparing physical	Comparing yourself physically with others	137	46.1	114	38.4	46	15.5	297	100
	Compared with other people’s physical appearance	198	66.7	88	29.6	11	3.7	297	100
Physical commenting	Being called a bad name	83	27.9	199	67.0	15	5.1	297	100
	Ridiculed, which leads to physical	146	49.2	136	45.8	15	5.1	297	100

The data on trigger factors for body shaming among adolescents highlights four primary influences. Media and pop culture contribute to 20.6% of body shaming experiences, underscoring the significant role of societal standards and representations in shaping adolescents’ self-perception. Peer pressure is the most prevalent factor, accounting for 29.7% of the cases, which emphasises the impact of social circles and the desire to fit in with peers. Family influence is closely related, with 30.0% of adolescents reporting it as a trigger, indicating that family attitudes and comments about appearance can profoundly affect young people’s body image. Lastly, personal insecurities, which account for 19.7% of the instances, reflect how internalised self-doubt and personal dissatisfaction can lead to or exacerbate experiences of body shaming (Table 4). These findings suggest that both external societal pressures and internal personal factors play crucial roles in the perpetuation of body shaming among adolescents.

**TABLE 4:** Trigger factors of body shaming.

Trigger factors	Frequency (*f* = 320)	%
Media and pop culture	66	20.6
Peer pressure	95	29.7
Family influence	96	30.0
Personal insecurities	63	19.7

*Source*: Syeda H, Shah I, Jan U, Mumtaz S. Exploring the impact of body shaming and emotional reactivity on the self-esteem of young adults. CARC Res Soc Sci. 2023;2(3):60–67. https://doi.org/10.58329/criss.v2i3.30

The data presented showcase the relationship between body shaming and stress among adolescents, analysed using Spearman’s rho correlation coefficient. The correlation coefficient between body shaming and stress is 0.404, indicating a moderate positive correlation. This means that as experiences of body shaming increase, levels of stress among adolescents also tend to rise. The significance level (Sig. 2-tailed) for this correlation is 0.000, which is well below the conventional threshold of 0.05, indicating that the correlation is statistically significant. The sample size for this analysis is 320, which provides a robust dataset for the correlation assessment (Table 5). These data underscore the significant impact body shaming has on stress levels, highlighting the psychological burden it places on adolescents.

**TABLE 5:** Bivariate analysis using the Spearman’s rank test.

Spearman’s rho	Body shaming	Stress
**Body shaming**		
Correlation coefficient	1.000	0.404**
Sig. (2-tailed)	-	0.000
*N*	320.000	320.000
**Stress**		
Correlation coefficient	0.404**	1.000
Sig. (2-tailed)	0.000	-
*N*	320.000	320.000

Sig., significance.

** , Correlation is significant at the 0.01 level (2-tailed).

## Discussion

### Key findings

This study on junior high school students at Jember, East Java, revealed that body shaming is a prevalent issue affecting adolescents, with significant psychological impacts. The study found that a considerable number of teenagers experience name-calling, gossip, criticism of speaking style, criticism for behaviour and fashion choices, and comparisons to others. Key trigger factors identified include family influence, peer pressure, media and personal insecurities. A significant positive correlation was found between body shaming and increased stress levels among adolescents.

### Discussion of key findings

The findings of this study closely align with previous research, demonstrating that body shaming is a pervasive issue among adolescents with significant negative impacts on their mental health.^4,5,13^ The high prevalence of body shaming observed in Jember mirrors global trends, indicating that this problem is not confined to larger metropolitan areas but is also prevalent in smaller communities. The study highlights the critical role of family influence in shaping adolescents’ body image perceptions, with family members, particularly parents and siblings, often being the first to impact how young people view themselves.^14^ Whether intentional or unintentional, comments about weight while in this study, BMI also affected susceptibility, especially among females and those with very low or normal BMIs, appearance or eating habits can profoundly affect adolescents’ self-esteem. Additionally, peer influence is identified as a significant factor, as the need for acceptance within social groups makes adolescents particularly vulnerable to the opinions and judgments of their peers. This pressure to conform to group norms regarding appearance often leads to increased stress and negative body image.

Moreover, the study underscores the impact of media and cultural norms, with the constant exposure to idealised body types and societal beauty standards significantly shaping adolescents’ perceptions of their bodies. In communities like Jember, cultural values can further reinforce these standards, making it difficult for adolescents to develop a positive body image if they do not conform to the cultural ideal. Overall, the study confirms the widespread nature of body shaming among adolescents and emphasises the complex interplay of family, peer, media and cultural influences that contribute to this issue.^9,15^

### Strengths and limitations

The study’s strengths include a comprehensive mixed methods approach, combining quantitative data with qualitative insights to provide a nuanced understanding of body shaming among adolescents. The use of validated instruments for data collection and a robust sample size enhances the reliability of the findings. However, the study’s cross-sectional design limits the ability to infer causality between body shaming and stress. The focus on junior high school students at Jember, East Java, may limit the generalisability of the findings to other regions or age groups. Additionally, potential biases in self-reported data, such as social desirability and recollection bias, must be considered when interpreting the results.

### Implications or recommendations

The study’s findings have important implications for parents, educators, policymakers and healthcare providers. There is a pressing need for comprehensive anti-bullying initiatives within educational institutions that specifically address body shaming, promote self-esteem and encourage body positivity among students. Training for educators and school counsellors to identify and address body shaming behaviours is crucial. Parental involvement in promoting healthy body image and self-acceptance at home is essential. Policymakers shall consider implementing regulations that mandate body image education in school curricula. Furthermore, societal reforms to promote inclusive beauty standards and accurate media representations of diverse body types are necessary to combat body shaming. Future research should employ longitudinal designs to explore causal relationships and investigate the moderating effects of variables such as socioeconomic status and personality traits. Integrating heterogeneous populations and expanding the range of mediating variables will provide a more comprehensive understanding of the factors driving body shaming and its impact.

## Conclusion

This study on body shaming among early adolescents attending junior high school in Jember, East Java, reveals that body shaming is a prevalent issue with significant mental health implications. Most respondents experienced moderate levels of body shaming, with age, gender and BMI influencing their susceptibility, particularly among females and those with very low or normal BMIs. The psychological toll includes increased stress, anxiety, depression and low self-esteem, highlighting the need for awareness and targeted interventions. Educators, counsellors and medical professionals must implement age- and gender-sensitive strategies, including nutritional education and counselling support.

While the study contributes valuable insights, limitations such as a small sample size, potential self-report bias and a cross-sectional design must be acknowledged. Future research should consider additional confounding variables and employ longitudinal designs to deepen our understanding. Despite these limitations, the findings underscore the urgent need for comprehensive strategies to support the mental health and well-being of this vulnerable population.
